# Imaza­lil: 1-[2-(2,4-dichloro­phen­yl)-2-(prop-2-en­yloxy)eth­yl]-1*H*-imidazole

**DOI:** 10.1107/S1600536811018241

**Published:** 2011-05-20

**Authors:** Sanghun Cheon, Yong Woon Shin, Ki-Min Park, Jineun Kim, Tae Ho Kim

**Affiliations:** a Department of Chemistry and Research Institute of Natural Sciences, Gyeongsang National University, Jinju 660-701, Republic of Korea; b Test & Analytical Laboratory, Korea Food & Drug Administration, 123-7 Yongdang-dong, Busan 608-829, Republic of Korea

## Abstract

In the title compound, C_14_H_14_Cl_2_N_2_O, the imidazole ring is almost parallel to the benzene ring, the dihedral angle between them being 7.3 (2)°. In the crystal, there is an inter­molecular C—Cl⋯π inter­action (Cl⋯centroid = 3.36 Å and C—Cl⋯centroid = 89.2°). In addition, a Cl⋯Cl contact of 3.411 (1) Å and an inter­molecular C—H⋯N hydrogen bond are observed. These inter­actions contribute to the stabilization of the crystal packing.

## Related literature

For information on the toxicity of the title compound, see: Sisman & Türkez (2010 ▶). For related structures, see: Bisaha *et al.* (2005 ▶).
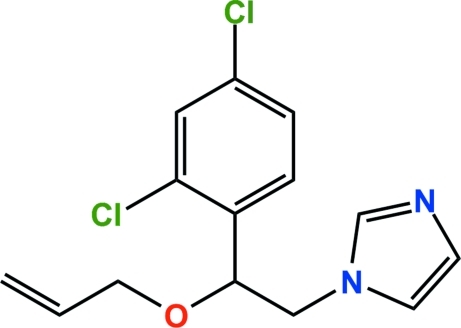


## Experimental

### 

#### Crystal data

C_14_H_14_Cl_2_N_2_O
*M* *_r_* = 297.17
Monoclinic, 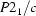

*a* = 7.9374 (6) Å
*b* = 13.4144 (12) Å
*c* = 13.479 (1) Å
β = 103.386 (5)°
*V* = 1396.19 (19) Å^3^
*Z* = 4
Mo *K*α radiation
μ = 0.46 mm^−1^
*T* = 173 K
0.20 × 0.09 × 0.08 mm

#### Data collection

Bruker APEXII CCD diffractometer
Absorption correction: multi-scan (*SADABS*; Sheldrick, 1996 ▶) *T*_min_ = 0.914, *T*_max_ = 0.964
12201 measured reflections
3040 independent reflections
2282 reflections with *I* > 2σ(*I*)
*R*_int_ = 0.069

#### Refinement

*R*[*F* ^2^ > 2σ(*F* ^2^)] = 0.046
*wR*(*F* ^2^) = 0.119
*S* = 1.08
3040 reflections
172 parameters
H-atom parameters constrained
Δρ_max_ = 0.37 e Å^−3^
Δρ_min_ = −0.27 e Å^−3^

### 

Data collection: *APEX2* (Bruker, 2006 ▶); cell refinement: *SAINT* (Bruker, 2006 ▶); data reduction: *SAINT*; program(s) used to solve structure: *SHELXTL* (Sheldrick, 2008 ▶); program(s) used to refine structure: *SHELXTL*; molecular graphics: *SHELXTL* and *DIAMOND* (Brandenburg, 1998 ▶); software used to prepare material for publication: *SHELXTL*.

## Supplementary Material

Crystal structure: contains datablocks global, I. DOI: 10.1107/S1600536811018241/wn2433sup1.cif

Structure factors: contains datablocks I. DOI: 10.1107/S1600536811018241/wn2433Isup2.hkl

Supplementary material file. DOI: 10.1107/S1600536811018241/wn2433Isup3.cml

Additional supplementary materials:  crystallographic information; 3D view; checkCIF report

## Figures and Tables

**Table 1:** Hydrogen-bond geometry (Å, °)

*D*—H⋯*A*	*D*—H	H⋯*A*	*D*⋯*A*	*D*—H⋯*A*
C4—H4⋯N2^i^	0.95	2.66	3.562 (3)	159

Symmetry code: (i) 
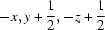
.

**Fig. 1.**
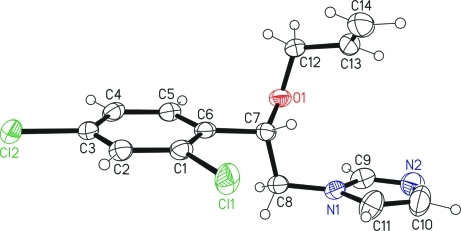
The molecular structure of the title compound. Displacement ellipsoids are drawn at the 50% probability level. H atoms are shown as small spheres of arbitrary radius.

**Fig. 2.**
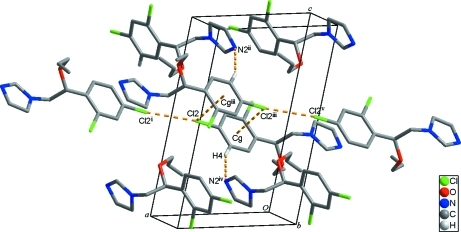
Crystal packing of the title compound with intermolecular C—H···N hydrogen bonds, C—Cl···π interactions and Cl···Cl contacts shown as dashed lines. H atoms not involved in intermolecular interactions have been omitted for clarity. [Symmetry codes: (i) -x + 2, -y + 2, -z + 1; (ii) x + 1, -y + 1.5, z + 1/2; (iii) -x + 1, -y + 2, -z + 1; (iv) -x, y + 1/2, -z + 1/2; (v) x - 1, y, z.]

## supplementary crystallographic information

### Comment

Imazalil (systematic name: 1-[2-(2,4-dichlorophenyl)-2- (2-propen-1-yloxy)ethyl]-1*H*-imidazole) is a fungicide widely used in agriculture, particularly in the growth of citrus fruits. It is also known as "enilconazole" (Sisman & Türkez, 2010). However,until now its crystal structure has not been reported.

In the title compound (Fig. 1), the imidazole ring is almost parallel to the benzene ring, the dihedral angle between them being 7.3 (2)°. All bond lengths and bond angles are normal and comparable to those observed in similar crystal structures (Bisaha *et al.* 2005).

In the crystal structure, as shown in Fig. 2, weak intermolecular C—H···N hydrogen bonds (Table 1) and a Cl···Cl contact of 3.411 (1) Å are observed. There are also C—Cl···π interactions involving chlorine Cl2 and benzene ring (C1–C6), with a Cl···centroid (*Cg*^iii^) distance of 3.36 Å and a C3—Cl2···*Cg*^iii^ angle of 89.2° [symmetry code: (iii) -*x* + 1, -*y* + 2, -*z* + 1] (Fig. 2). These intermolecular interactions may contribute to the stabilization of the crystal packing.

### Experimental

The title compound was purchased from the Dr. Ehrenstorfer GmbH Company. Slow evaporation of a solution in CH_2_Cl_2_ gave single crystals suitable for X-ray analysis.

### Refinement

The H atoms were geometrically positioned and refined as riding. C—H = 0.95 Å for Csp^2^, C—H = 0.99 Å for methylene C and C—H = 1.00 Å for methine C; *U*_iso_(H) = 1.2*U*_eq_(parent atom).

### Crystal data

**Table:**

C_14_H_14_Cl_2_N_2_O	*F*(000) = 616
*M**_r_* = 297.17	*D*_x_ = 1.414 Mg m^−^^3^
Monoclinic, *P*2_1_/*c*	Mo *K*α radiation, λ = 0.71073 Å
Hall symbol: -P 2ybc	Cell parameters from 2420 reflections
*a* = 7.9374 (6) Å	θ = 2.2–28.4°
*b* = 13.4144 (12) Å	µ = 0.46 mm^−^^1^
*c* = 13.479 (1) Å	*T* = 173 K
β = 103.386 (5)°	Plate, colourless
*V* = 1396.19 (19) Å^3^	0.20 × 0.09 × 0.08 mm
*Z* = 4	

### Data collection

**Table:**

Bruker APEXII CCD diffractometer	3040 independent reflections
Radiation source: fine-focus sealed tube	2282 reflections with *I* > 2σ(*I*)
graphite	*R*_int_ = 0.069
φ and ω scans	θ_max_ = 27.0°, θ_min_ = 2.2°
Absorption correction: multi-scan (*SADABS*; Sheldrick, 1996)	*h* = −9→10
*T*_min_ = 0.914, *T*_max_ = 0.964	*k* = −17→14
12201 measured reflections	*l* = −17→17

### Refinement

**Table:**

Refinement on *F*^2^	Primary atom site location: structure-invariant direct methods
Least-squares matrix: full	Secondary atom site location: difference Fourier map
*R*[*F*^2^ > 2σ(*F*^2^)] = 0.046	Hydrogen site location: inferred from neighbouring sites
*wR*(*F*^2^) = 0.119	H-atom parameters constrained
*S* = 1.08	*w* = 1/[σ^2^(*F*_o_^2^) + (0.0525*P*)^2^ + 0.2498*P*] where *P* = (*F*_o_^2^ + 2*F*_c_^2^)/3
3040 reflections	(Δ/σ)_max_ < 0.001
172 parameters	Δρ_max_ = 0.37 e Å^−^^3^
0 restraints	Δρ_min_ = −0.27 e Å^−^^3^

### Special details

**Table:**

Geometry. All e.s.d.'s (except the e.s.d. in the dihedral angle between two l.s. planes) are estimated using the full covariance matrix. The cell e.s.d.'s are taken into account individually in the estimation of e.s.d.'s in distances, angles and torsion angles; correlations between e.s.d.'s in cell parameters are only used when they are defined by crystal symmetry. An approximate (isotropic) treatment of cell e.s.d.'s is used for estimating e.s.d.'s involving l.s. planes.
Refinement. Refinement of *F*^2^ against ALL reflections. The weighted *R*-factor *wR* and goodness of fit *S* are based on *F*^2^, conventional *R*-factors *R* are based on *F*, with *F* set to zero for negative *F*^2^. The threshold expression of *F*^2^ > σ(*F*^2^) is used only for calculating *R*-factors(gt) *etc*. and is not relevant to the choice of reflections for refinement. *R*-factors based on *F*^2^ are statistically about twice as large as those based on *F*, and *R*- factors based on ALL data will be even larger.

### Fractional atomic coordinates and isotropic or equivalent isotropic displacement parameters (Å^2^)

**Table:**

	*x*	*y*	*z*	*U*_iso_*/*U*_eq_	
Cl1	0.41595 (8)	0.69956 (5)	0.57733 (5)	0.0410 (2)	
Cl2	0.77994 (7)	0.98990 (4)	0.47431 (5)	0.03569 (19)	
O1	0.0431 (2)	0.71310 (11)	0.25901 (12)	0.0294 (4)	
N1	−0.1440 (2)	0.69576 (14)	0.40437 (15)	0.0289 (4)	
N2	−0.3936 (3)	0.60867 (17)	0.34432 (19)	0.0465 (6)	
C1	0.4327 (3)	0.78309 (16)	0.48568 (17)	0.0282 (5)	
C2	0.5822 (3)	0.84344 (16)	0.51200 (18)	0.0285 (5)	
H2	0.6668	0.8363	0.5742	0.034*	
C3	0.5937 (3)	0.91175 (16)	0.44116 (18)	0.0279 (5)	
C4	0.4636 (3)	0.91953 (16)	0.34627 (18)	0.0297 (5)	
H4	0.4775	0.9689	0.2982	0.036*	
C5	0.3172 (3)	0.85669 (16)	0.32241 (18)	0.0277 (5)	
H5	0.2343	0.8632	0.2594	0.033*	
C6	0.2977 (3)	0.78727 (15)	0.39102 (17)	0.0255 (5)	
C7	0.1325 (3)	0.72205 (16)	0.36550 (17)	0.0253 (5)	
H7	0.1605	0.6540	0.3949	0.030*	
C8	−0.0025 (3)	0.76633 (17)	0.40885 (19)	0.0311 (5)	
H8A	0.0469	0.7854	0.4806	0.037*	
H8B	−0.0474	0.8274	0.3704	0.037*	
C9	−0.3009 (3)	0.68361 (19)	0.3287 (2)	0.0377 (6)	
H9	−0.3345	0.7269	0.2718	0.045*	
C10	−0.2883 (4)	0.5710 (2)	0.4355 (2)	0.0505 (7)	
H10	−0.3197	0.5139	0.4688	0.061*	
C11	−0.1353 (4)	0.6230 (2)	0.4733 (2)	0.0440 (6)	
H11	−0.0471	0.6102	0.5328	0.053*	
C12	0.1149 (3)	0.63442 (17)	0.21546 (18)	0.0312 (5)	
H12A	0.2426	0.6410	0.2344	0.037*	
H12B	0.0777	0.6401	0.1403	0.037*	
C13	0.0682 (3)	0.53464 (18)	0.24568 (19)	0.0350 (6)	
H13	−0.0508	0.5167	0.2311	0.042*	
C14	0.1862 (4)	0.4672 (2)	0.2933 (2)	0.0451 (7)	
H14B	0.3060	0.4833	0.3087	0.054*	
H14A	0.1493	0.4037	0.3112	0.054*	

### Atomic displacement parameters (Å^2^)

**Table:**

	*U*^11^	*U*^22^	*U*^33^	*U*^12^	*U*^13^	*U*^23^
Cl1	0.0519 (4)	0.0404 (4)	0.0294 (3)	−0.0131 (3)	0.0066 (3)	0.0096 (3)
Cl2	0.0339 (3)	0.0338 (3)	0.0442 (4)	−0.0075 (2)	0.0189 (3)	−0.0031 (3)
O1	0.0340 (8)	0.0319 (8)	0.0244 (8)	0.0010 (7)	0.0111 (7)	−0.0023 (7)
N1	0.0320 (10)	0.0296 (10)	0.0285 (10)	0.0003 (8)	0.0139 (9)	−0.0005 (8)
N2	0.0390 (12)	0.0524 (14)	0.0515 (15)	−0.0104 (11)	0.0170 (11)	−0.0142 (12)
C1	0.0376 (12)	0.0253 (11)	0.0253 (12)	0.0009 (10)	0.0147 (10)	0.0020 (9)
C2	0.0310 (12)	0.0305 (12)	0.0256 (12)	0.0001 (10)	0.0098 (10)	−0.0012 (10)
C3	0.0286 (11)	0.0249 (11)	0.0347 (13)	−0.0021 (9)	0.0168 (10)	−0.0065 (10)
C4	0.0362 (12)	0.0251 (11)	0.0337 (13)	0.0022 (9)	0.0203 (11)	0.0035 (10)
C5	0.0305 (12)	0.0288 (12)	0.0256 (12)	0.0033 (9)	0.0104 (10)	0.0002 (10)
C6	0.0315 (12)	0.0233 (11)	0.0253 (11)	0.0030 (9)	0.0142 (10)	−0.0022 (9)
C7	0.0313 (11)	0.0236 (11)	0.0225 (11)	−0.0007 (9)	0.0090 (10)	−0.0019 (9)
C8	0.0399 (13)	0.0259 (12)	0.0324 (13)	−0.0017 (10)	0.0183 (11)	−0.0032 (10)
C9	0.0362 (13)	0.0440 (14)	0.0357 (14)	0.0031 (11)	0.0136 (12)	−0.0004 (12)
C10	0.0503 (16)	0.0398 (15)	0.066 (2)	−0.0085 (13)	0.0237 (16)	0.0085 (14)
C11	0.0441 (15)	0.0475 (15)	0.0410 (15)	−0.0023 (12)	0.0109 (13)	0.0163 (13)
C12	0.0367 (13)	0.0334 (12)	0.0277 (12)	−0.0062 (10)	0.0159 (11)	−0.0058 (10)
C13	0.0403 (13)	0.0357 (13)	0.0319 (13)	−0.0111 (11)	0.0143 (11)	−0.0077 (11)
C14	0.0631 (18)	0.0383 (14)	0.0387 (15)	−0.0040 (13)	0.0219 (14)	−0.0038 (12)

### Geometric parameters (Å, °)

**Table:**

Cl1—C1	1.696 (2)	C5—H5	0.9500
Cl2—C3	1.783 (2)	C6—C7	1.547 (3)
O1—C12	1.392 (3)	C7—C8	1.461 (3)
O1—C7	1.452 (3)	C7—H7	1.0000
N1—C11	1.338 (3)	C8—H8A	0.9900
N1—C9	1.425 (3)	C8—H8B	0.9900
N1—C8	1.459 (3)	C9—H9	0.9500
N2—C9	1.291 (3)	C10—C11	1.391 (4)
N2—C10	1.410 (4)	C10—H10	0.9500
C1—C2	1.412 (3)	C11—H11	0.9500
C1—C6	1.465 (3)	C12—C13	1.471 (3)
C2—C3	1.342 (3)	C12—H12A	0.9900
C2—H2	0.9500	C12—H12B	0.9900
C3—C4	1.450 (3)	C13—C14	1.353 (4)
C4—C5	1.411 (3)	C13—H13	0.9500
C4—H4	0.9500	C14—H14B	0.9500
C5—C6	1.346 (3)	C14—H14A	0.9500
			
C12—O1—C7	108.98 (17)	C6—C7—H7	109.2
C11—N1—C9	108.0 (2)	N1—C8—C7	110.39 (18)
C11—N1—C8	121.9 (2)	N1—C8—H8A	109.6
C9—N1—C8	129.8 (2)	C7—C8—H8A	109.6
C9—N2—C10	100.1 (2)	N1—C8—H8B	109.6
C2—C1—C6	126.8 (2)	C7—C8—H8B	109.6
C2—C1—Cl1	113.51 (18)	H8A—C8—H8B	108.1
C6—C1—Cl1	119.65 (16)	N2—C9—N1	114.1 (2)
C3—C2—C1	113.9 (2)	N2—C9—H9	122.9
C3—C2—H2	123.1	N1—C9—H9	122.9
C1—C2—H2	123.1	C11—C10—N2	115.5 (2)
C2—C3—C4	121.7 (2)	C11—C10—H10	122.2
C2—C3—Cl2	114.37 (18)	N2—C10—H10	122.2
C4—C3—Cl2	123.96 (17)	N1—C11—C10	102.2 (2)
C5—C4—C3	122.6 (2)	N1—C11—H11	128.9
C5—C4—H4	118.7	C10—C11—H11	128.9
C3—C4—H4	118.7	O1—C12—C13	114.76 (18)
C6—C5—C4	118.4 (2)	O1—C12—H12A	108.6
C6—C5—H5	120.8	C13—C12—H12A	108.6
C4—C5—H5	120.8	O1—C12—H12B	108.6
C5—C6—C1	116.6 (2)	C13—C12—H12B	108.6
C5—C6—C7	117.7 (2)	H12A—C12—H12B	107.6
C1—C6—C7	125.69 (19)	C14—C13—C12	123.3 (2)
O1—C7—C8	101.13 (18)	C14—C13—H13	118.4
O1—C7—C6	117.51 (17)	C12—C13—H13	118.4
C8—C7—C6	110.10 (17)	C13—C14—H14B	120.0
O1—C7—H7	109.2	C13—C14—H14A	120.0
C8—C7—H7	109.2	H14B—C14—H14A	120.0
			
C6—C1—C2—C3	1.4 (3)	C1—C6—C7—O1	−160.79 (18)
Cl1—C1—C2—C3	−178.10 (16)	C5—C6—C7—C8	−93.1 (2)
C1—C2—C3—C4	−1.0 (3)	C1—C6—C7—C8	84.2 (3)
C1—C2—C3—Cl2	179.00 (15)	C11—N1—C8—C7	80.7 (3)
C2—C3—C4—C5	0.3 (3)	C9—N1—C8—C7	−93.6 (3)
Cl2—C3—C4—C5	−179.78 (16)	O1—C7—C8—N1	67.1 (2)
C3—C4—C5—C6	0.4 (3)	C6—C7—C8—N1	−167.88 (18)
C4—C5—C6—C1	−0.1 (3)	C10—N2—C9—N1	−0.1 (3)
C4—C5—C6—C7	177.48 (18)	C11—N1—C9—N2	0.2 (3)
C2—C1—C6—C5	−0.8 (3)	C8—N1—C9—N2	175.2 (2)
Cl1—C1—C6—C5	178.64 (16)	C9—N2—C10—C11	0.0 (3)
C2—C1—C6—C7	−178.2 (2)	C9—N1—C11—C10	−0.2 (3)
Cl1—C1—C6—C7	1.3 (3)	C8—N1—C11—C10	−175.6 (2)
C12—O1—C7—C8	−152.67 (17)	N2—C10—C11—N1	0.2 (3)
C12—O1—C7—C6	87.5 (2)	C7—O1—C12—C13	73.9 (2)
C5—C6—C7—O1	21.8 (3)	O1—C12—C13—C14	−120.4 (3)

### Hydrogen-bond geometry (Å, °)

**Table:**

*D*—H···*A*	*D*—H	H···*A*	*D*···*A*	*D*—H···*A*
C4—H4···N2^i^	0.95	2.66	3.562 (3)	159

Symmetry codes: (i) −*x*, *y*+1/2, −*z*+1/2.
